# Virology under the Microscope—a Call for Rational Discourse

**DOI:** 10.1128/mbio.00188-23

**Published:** 2023-01-26

**Authors:** Felicia Goodrum, Anice C. Lowen, Seema Lakdawala, James Alwine, Arturo Casadevall, Michael J. Imperiale, Walter Atwood, Daphne Avgousti, Joel Baines, Bruce Banfield, Lawrence Banks, Sumita Bhaduri-McIntosh, Deepta Bhattacharya, Daniel Blanco-Melo, David Bloom, Adrianus Boon, Steeve Boulant, Curtis Brandt, Andrew Broadbent, Christopher Brooke, Craig Cameron, Samuel Campos, Patrizia Caposio, Gary Chan, Anna Cliffe, John Coffin, Kathleen Collins, Blossom Damania, Matthew Daugherty, Kari Debbink, James DeCaprio, Terence Dermody, Jimmy Dikeakos, Daniel DiMaio, Rhoel Dinglasan, W. Paul Duprex, Rebecca Dutch, Nels Elde, Michael Emerman, Lynn Enquist, Bentley Fane, Ana Fernandez-Sesma, Michelle Flenniken, Lori Frappier, Matthew Frieman, Klaus Frueh, Michaela Gack, Marta Gaglia, Tom Gallagher, Denise Galloway, Adolfo García-Sastre, Adam Geballe, Britt Glaunsinger, Stephen Goff, Alexander Greninger, Meaghan Hancock, Eva Harris, Nicholas Heaton, Mark Heise, Ekaterina Heldwein, Brenda Hogue, Stacy Horner, Edward Hutchinson, Joseph Hyser, William Jackson, Robert Kalejta, Jeremy Kamil, Stephanie Karst, Frank Kirchhoff, David Knipe, Timothy Kowalik, Michael Lagunoff, Laimonis Laimins, Ryan Langlois, Adam Lauring, Benhur Lee, David Leib, Shan-Lu Liu, Richard Longnecker, Carolina Lopez, Micah Luftig, Jennifer Lund, Balaji Manicassamy, Grant McFadden, Michael McIntosh, Andrew Mehle, W. Allen Miller, Ian Mohr, Cary Moody, Nathaniel Moorman, Anne Moscona, Bryan Mounce, Joshua Munger, Karl Münger, Eain Murphy, Mojgan Naghavi, Jay Nelson, Christopher Neufeldt, Janko Nikolich, Christine O'Connor, Akira Ono, Walter Orenstein, David Ornelles, Jing-hsiung Ou, John Parker, Colin Parrish, Andrew Pekosz, Philip Pellett, Julie Pfeiffer, Richard Plemper, Stephen Polyak, John Purdy, Dohun Pyeon, Miguel Quinones-Mateu, Rolf Renne, Charles Rice, John Schoggins, Richard Roller, Charles Russell, Rozanne Sandri-Goldin, Martin Sapp, Luis Schang, Scott Schmid, Stacey Schultz-Cherry, Bert Semler, Thomas Shenk, Guido Silvestri, Viviana Simon, Gregory Smith, Jason Smith, Katherine Spindler, Megan Stanifer, Kanta Subbarao, Wesley Sundquist, Mehul Suthar, Troy Sutton, Andrew Tai, Vera Tarakanova, Benjamin tenOever, Scott Tibbetts, Stephen Tompkins, Zsolt Toth, Koenraad van Doorslaer, Marco Vignuzzi, Nicholas Wallace, Derek Walsh, Michael Weekes, Jason Weinberg, Matthew Weitzman, Sandra Weller, Sean Whelan, Elizabeth White, Bryan Williams, Christiane Wobus, Scott Wong, Andrew Yurochko

**Affiliations:** a Department of Immunobiology, BIO5 Institute, University of Arizona, Tucson, Arizona, USA; b Department of Microbiology and Immunology, Emory University School of Medicine, Atlanta, Georgia, USA; c Department of Cancer Biology, University of Pennsylvania, Philadelphia, Pennsylvania, USA; d Department of Molecular Microbiology and Immunology, Johns Hopkins Bloomberg School of Public Health, Baltimore, Maryland, USA; e Department of Microbiology and Immunology, University of Michigan, Ann Arbor, Michigan, USA; f Brown University, Providence, Rhode Island, USA; g Fred Hutchinson Cancer Research Center, Seattle, Washington, USA; h Cornell University, Ithaca, New York, USA; i Queen's University, Kingston, Ontario, Canada; j International Centre for Genetic Engineering and Biotechnology, Trieste, Italy; k University of Florida, Gainesville, Florida, USA; l University of Arizona, Tucson, Arizona, USA; m Washington University, St. Louis, Missouri, USA; n University of Wisconsin—Madison, Madison, Wisconsin, USA; o University of Maryland, College Park, Maryland, USA; p University of Illinois at Urbana-Champaign, Urbana, Illinois, USA; q University of North Carolina, Chapel Hill, North Carolina, USA; r Oregon Health and Science University, Beaverton, Oregon, USA; s SUNY Upstate Medical University, Syracuse, New York, USA; t University of Virginia, Charlottesville, Virginia, USA; u Tufts University, Boston, Massachusetts, USA; v University of Michigan, Ann Arbor, Michigan, USA; w University of California, San Diego, La Jolla, California, USA; x Johns Hopkins University, Baltimore, Maryland, USA; y Harvard Medical School, Boston, Massachusetts, USA; z University of Pittsburgh, Pittsburgh, Pennsylvania, USA; aa Western University, London, Ontario, Canada; bb Yale University, New Haven, Connecticut, USA; cc University of Kentucky, Lexington, Kentucky, USA; dd University of Utah, Salt Lake City, Utah, USA; ee Princeton University, Princeton, New Jersey, USA; ff Icahn School of Medicine at Mount Sinai, New York, New York, USA; gg Montana State University, Bozeman, Montana, USA; hh University of Toronto, Toronto, Ontario, Canada; ii Florida Research and Innovation Center, Port Saint Lucie, Florida, USA; jj Loyola University, Maywood, Illinois, USA; kk University of California, Berkeley, Berkeley, California, USA; ll Columbia University, New York, New York, USA; mm University of Washington, Seattle, Washington, USA; nn Duke University, Durham, North Carolina, USA; oo Arizona State University, Tempe, Arizona, USA; pp MRC-University of Glasgow, Glasgow, United Kingdom; qq Baylor University, Houston, Texas, USA; rr Louisiana State University, Shreveport, Louisiana, USA; ss Ulm University, Ulm, Germany; tt University of Massachusetts, Worcester, Massachusetts, USA; uu Northwestern University, Chicago, Illinois, USA; vv University of Minnesota, Minneapolis, Minnesota, USA; ww Dartmouth College, Lebanon, New Hampshire, USA; xx The Ohio State University, Columbus, Ohio, USA; yy University of Iowa, Iowa City, Iowa, USA; zz Iowa State University, Ames, Iowa, USA; aaa New York University, New York, New York, USA; bbb University of Rochester, Rochester, New York, USA; ccc Emory University, Atlanta, Georgia, USA; ddd Cleveland Clinic, Cleveland, Ohio, USA; eee Wake Forest University, Winston-Salem, North Carolina, USA; fff University of Southern California, Los Angeles, California, USA; ggg Wayne State University, Detroit, Michigan, USA; hhh University of Texas, Dallas, Texas, USA; iii Georgia State University, Atlanta, Georgia, USA; jjj Michigan State University, East Lansing, Michigan, USA; kkk The Rockefeller University, New York, New York, USA; lll St. Jude Children's Research Hospital, Memphis, Tennessee, USA; mmm University of California, Irvine, Irvine, California, USA; nnn University of Colorado, Boulder, Colorado, USA; ooo The Peter Doherty Institute, Melbourne, Victoria, Australia; ppp The Pennsylvania State University, University Park, Pennsylvania, USA; qqq Medical College of Wisconsin, Milwaukee, Wisconsin, USA; rrr University of Georgia, Athens, Georgia, USA; sss A*STAR Infectious Diseases Labs, Singapore; ttt Kansas State University, Manhattan, Kansas, USA; uuu University of Cambridge, Cambridge, United Kingdom; vvv University of Pennsylvania, Philadelphia, Pennsylvania, USA; www University of Connecticut, Farmington, Connecticut, USA; xxx Monash University, Clayton, Victoria, Australia

**Keywords:** COVID-19, Coronavirus, DURC, Gain of function, SARS-CoV-2, biosafety, influenza, pandemic, vaccines, zoonosis

## Abstract

Viruses have brought humanity many challenges: respiratory infection, cancer, neurological impairment and immunosuppression to name a few. Virology research over the last 60+ years has responded to reduce this disease burden with vaccines and antivirals. Despite this long history, the COVID-19 pandemic has brought unprecedented attention to the field of virology. Some of this attention is focused on concern about the safe conduct of research with human pathogens. A small but vocal group of individuals has seized upon these concerns – conflating legitimate questions about safely conducting virus-related research with uncertainties over the origins of SARS-CoV-2. The result has fueled public confusion and, in many instances, ill-informed condemnation of virology. With this article, we seek to promote a return to rational discourse. We explain the use of gain-of-function approaches in science, discuss the possible origins of SARS-CoV-2 and outline current regulatory structures that provide oversight for virological research in the United States. By offering our expertise, we – a broad group of working virologists – seek to aid policy makers in navigating these controversial issues. Balanced, evidence-based discourse is essential to addressing public concern while maintaining and expanding much-needed research in virology.

## COMMENTARY

Just 30,000 nucleotides of single-stranded RNA, neatly packaged as a coronavirus, brought the world to its knees socially, economically, ethically, and morally during the COVID-19 pandemic. COVID-19 has cast a harsh light on the many cracks, fissures and disparities in our public health system, and the inability to broadly come together to face a colossal crisis and focus on the needs of the most vulnerable. However, scientists worked together and responded to the threat with impressive speed, drawing on critical previous research on coronaviruses and other viral systems. Virologists, immunologists and microbiologists from around the globe collaborated together with scientists from allied disciplines, such as infectious diseases and epidemiology. They confronted the virus through research to understand its pathogenesis and transmission, through surveillance to track the emergence of variants, and through the development of rapid tests, vaccines, antivirals and monoclonal antibodies. The SARS-CoV-2 pandemic would have claimed a substantially larger number of lives and caused more economic disruption were it not for this unprecedented collaborative scientific response. Nevertheless, the SARS-CoV-2 pandemic has also brought virology under the microscope with concerns about safety of virology research and the uncertainties around the origins of SARS-CoV-2. Here we provide an evidence- based discourse to address key issues.

### Virology research under scrutiny.

Congress has a constitutional mandate to provide oversight to federally funded research. As a new Congress convenes in the United States, there is an opportunity for oversight hearings related to research in virology and the virology community stands ready to partner with Congress and lend our expertise. Our hope is that these hearings will highlight the enormous contributions of virology, including gain-of-function experiments, to human health (Table 1). However, we fear that some may use any such hearings to discredit virology and virologists and – whether intentional or not – add fuel to an anti-science, fear-based movement. Should such hearings lead to Congress legislating restrictions on scientific research, the outcome could impede our ability to predict, prepare, and respond to emerging viral threats. An equally devastating outcome would be to sow even more public distrust in science, which would limit our ability to confront viruses in general and increase the human burden from viral diseases.

**TABLE 1:** Human viral diseases for which virology research has delivered vaccines and antiviral drugs

Disease	Vaccine	Antiviral
Adenovirus	Yes	No
AIDS	No	Yes
Cervical and Head/neck Cancer	Yes	No
COVID-19	Yes	Yes
Ebola virus	Yes	Yes
Japanese encephalitis	Yes	No
Hepatitis A	Yes	No
Hepatitis B	Yes	Yes
Hepatitis C	No	Yes
Herpes (HSV and CMV)	No	Yes
Influenza	Yes	Yes
Measles	Yes	No
Mpox	Yes	Yes
Mumps	Yes	No
Polio	Yes	No
Rabies	Yes	No
Respiratory syncytial virus	No	Yes
Rotavirus	Yes	No
Rubella	Yes	No
Smallpox	Yes	Yes
Tick borne encephalitis	Yes	No
Yellow fever	Yes	No
Varicella and zoster	Yes	Yes

### The origin of SARS-CoV-2.

A major point of contention in discussions of the COVID-19 pandemic has been the origin of SARS-CoV-2, with two major camps arguing that the virus either originated from animal-to-human transmission (zoonosis) or by a laboratory leak (1–3). Most virologists have been open-minded about the possible origins of SARS-CoV-2 and have formed opinions based on the best available evidence, as is done for all scientific questions (4). While each of these possibilities is plausible and has been investigated, currently the zoonosis hypothesis has the strongest supporting evidence (5–8). Zoonosis involves transmission of the virus as a consequence of close proximity between humans and wild animals, a scenario that has occurred repeatedly over time, leading to the emergence of many viruses, including Ebola virus, other coronaviruses, influenza A virus, mpox virus, and others (9–11). The lab-origin hypothesis suggests an accident at best or nefarious actors at the worst. At this time and based on the available data, there is no compelling evidence to support either of these lab-origin scenarios. It is important that scientists, the public, and public figures follow the evidence and limit speculation that can become fodder for misinformation and conspiracy theories. For example, on January 1, 2023, *USA Today* published an article discussing where the next pandemic could originate and disproportionately emphasized risk from manmade threats and lab accidents while minimizing the fact that most pandemics are zoonoses and never mentioning how virological research could mitigate risk (12). Unfounded accusations of a lab leak event or nefarious research in Chinese laboratories will hasten the deterioration of important partnerships between the US and China that are critical for early detection and preparedness for seasonal influenza and future pandemics.

### Gain-of-function research.

Despite the paucity of evidence for a laboratory-origin of SARS-CoV-2, discussion of this possibility has driven a second controversy to the forefront of science policy discussion: the use of gain-of-function approaches in virology. Although the phrase ‘gain-of-function’ is very problematic and inexact, it is commonly used, and we will use it here cautioning all its limitations (13). The source of concern in this area is that changing a virus to add new functionality may yield a dangerous pathogen. It is important to understand, however, that gain-of-function approaches incorporate a large proportion of *all* research because they are a powerful genetic tool in the laboratory. These include the development of cancer therapeutics, bacterial strategies for bioremediation, and the engineering of drought- or pest-resistant crops (Table 2). For example, some oncolytic viruses used to treat cancer mediate their effects because, using gain-of-function approaches, they have been endowed with new properties that kill tumors. At least two FDA-approved products resulted from providing viruses with new functions (Table 2). Gain-of-function research with pathogens of pandemic potential established that avian influenza viruses have the capacity to acquire mammalian transmissibility and that bat-associated coronaviruses posed a danger to humans years before COVID-19 (Table 2).

**TABLE 2:** Examples of useful gain-of-function experiments

Goal/result	Microbe	Gain-of-Function	Reference
Insect control	Baculovirus	Scorpion neurotoxin	(18)
Solid tumor therapy	Vaccinia	GM-CSF Expression	(19)^*a*^
Melanoma therapy	Herpes Simplex	GM-CSF Expression	(20)
COVID-19 vaccine	Adenovirus type 26	Expression of SARS-CoV-2 spike protein	(21)^*a*^
Repair Cardiac pacemaker	Adenovirus	Expression of sinoatrial node transcription factor	(22)
Treatment of bacterial infectious diseases	Bacteriophages	Expression of various payloads to enhance activity	(23)
Treating Citrus tree greening disease	Citrus tristeza virus	Spinach Defensin expression	(24)
Enhanced Lithium Batteries	E4 and M13 bacteriophage	Modified coat protein for carbon nanotube and cation binding	(25)
Rabbit control through immunocontraception	Myxoma virus	Expression of rabbit zona pellucida glycoproteins	(26)^*b*^
Mouse control through immunocontraception	Ectromelia	Expression of mouse zona pellucida glycoproteins	(27)^*b*^
Faster computers	M13 bacteriophage	Increased electrical conductance	(28)
Established that H5N1 has capacity for mammalian transmissibility	H5N1	Mutations leading to mammalian transmission	(29, 30)
Established danger from bat SARS-like coronavirus to humans	SARS-CoV	Bat coronavirus spike protein	(31)
Drought and salt resistance in plants	*Arabidopsis*	Over expression of vacuolar H+-ATPase	(32)
Resistance to dengue virus to reduce transmission	Mosquitoes	Transgenic expression of antibody to dengue virus	(33)
Resistance to freezing	Many species from plant to animals	Expression of anti-freeze proteins	(34)
Increase nitrogen fixation to reduce fertilizer need	Klebsiella variicola	122-fold increase in nitrogen fixation genes	(35)
Develop a new vaccine against cryptococcosis	Cryptococcus neoformans	Expression of gamma- interferon	(36)
Hormones for human therapy (e.g. insulin)	E. coli	Synthesis of human hormone (e.g. insulin)	(37)
Enzymes for food prepn such as pectinases for improved juice production	Yeast species	Enzyme expression for industrial use	(38)
CAR T cells	Lentivirus	Cancer immunotherapy	(39)
Dengue vaccine	Dengue/yellow fever virus	Recombinant DNA technology replaces genetic sequences in the yellow fever vaccine with dengue virus sequences	(40)^*b*^

a FDA approved product.

b Experiments done in viruses that classify as pathogens with pandemic potential.

**FIG 1:**
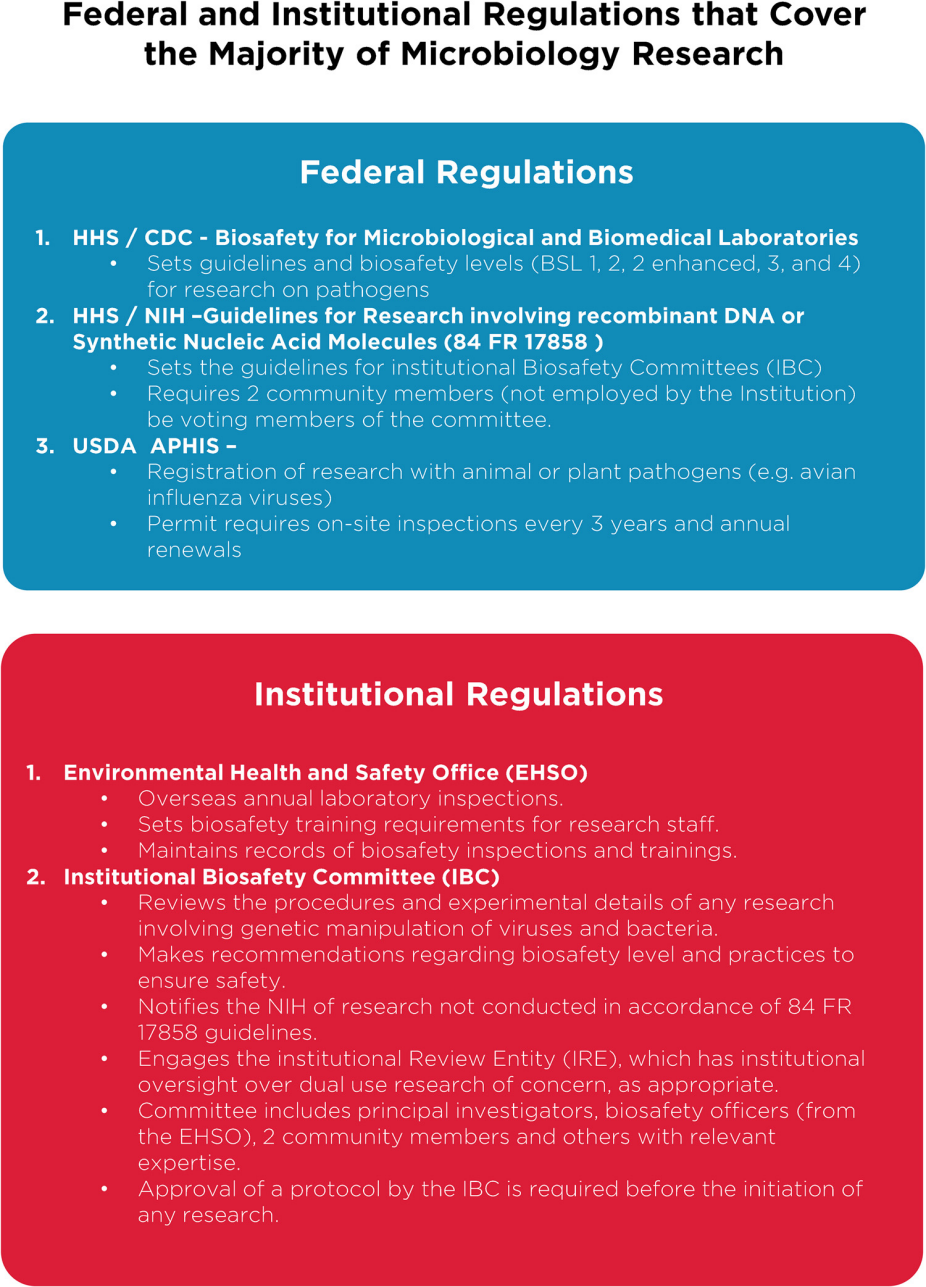
Federal and institutional regulations. A brief breakdown of current U.S. Department of Health and Human Services regulations on microbiology research and the implementation of federal requirements by individual institutions, through various committees and processes. Important additional oversight that is not shown includes that provided by occupational health services to ensure safety of research personnel, and federal and institutional regulation of research involving vertebrate animals or human subjects, which are overseen by Institutional Animal Care and Use Committees (IACUC; animal research) and Institutional Review Boards (IRB; human subjects research).

**FIG 2:**
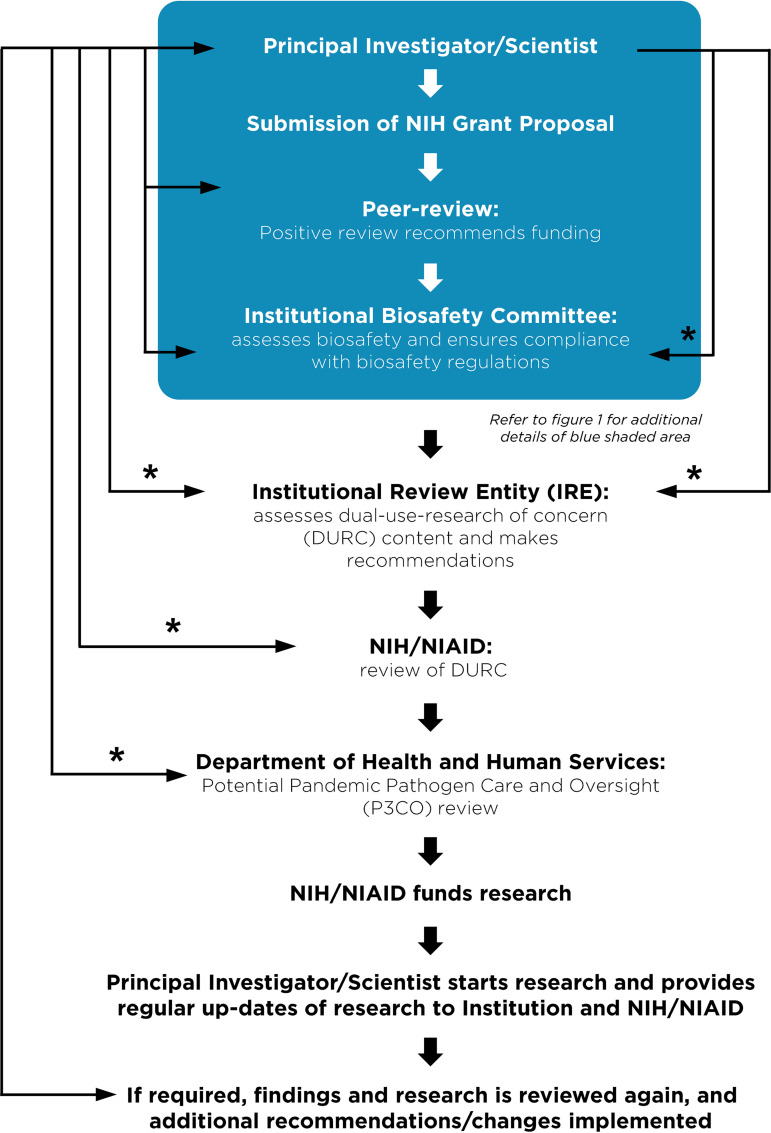
Current regulations surrounding funding, monitoring and approval of gain-of-function research of concern. A flow chart describing the steps a principal investigator must follow prior to receiving funding or initiating research considered dual-use-research-of-concern (DURC) on pathogens of pandemic potential (P3). The blue shaded box corresponds to the general practices that all investigators carry out at the institutional level described in Fig. 1. Research with the potential to enhance the properties of pathogen is referred for additional oversight. Asterisks (*) indicate an iterative process typically involving 1–3 reviews and revisions.

**FIG 3:**
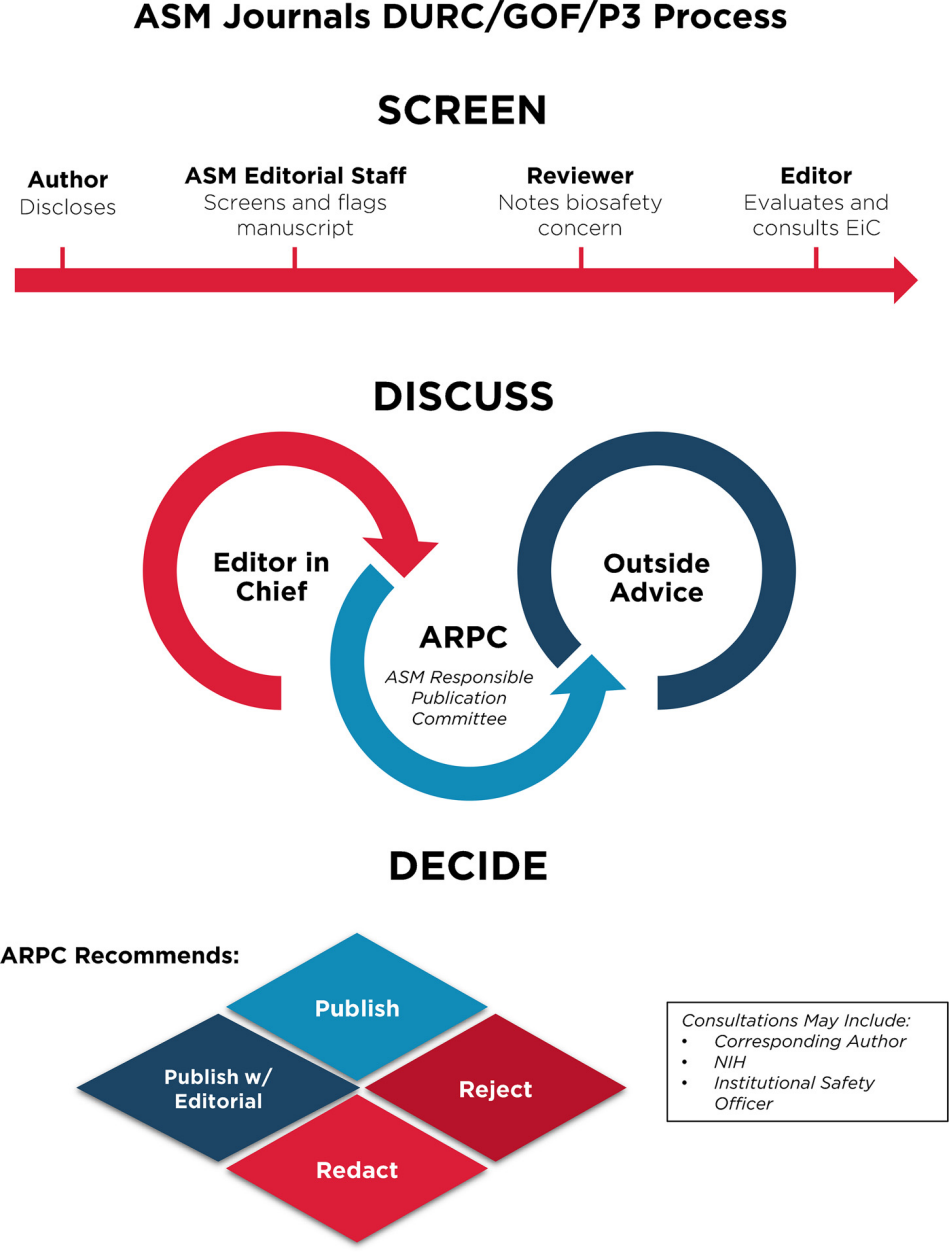
Publishing process for articles involving gain-of-function research-of-concern or with pathogens of pandemic potential at ASM journals. Articles in the GOF/DURC/P3 category are flagged by multiple layers at the screening stage. This process will initiate discussion among the scientific editor in chief and other subject matter experts and lead to a publication decision after careful consideration of risks and benefits.

Despite these clear benefits, a narrative has developed suggesting that gain-of-function research is synonymous with high-risk or nefarious activity to engineer or enhance pandemic pathogens. In truth, gain-of-function research is a valuable experimental approach that virologists use to address essential questions. Virologists do not take their work lightly and thoughtfully propose experiments to address essential questions. Virologists do not operate in isolation to judge the risks of experiments: layers of regulation are in place such that risks are considered by individuals with diverse perspectives and expertise (Fig. 1). The vast majority of virology experiments could not enhance pandemic potential (referred to in the United States as gain-of-function research-of-concern). Those rare experiments that could are currently subject to stringent oversight through the U.S. Government under programs known as dual-use- research-of-concern (DURC) (14) and potential-pandemic-pathogens-care-and-oversight (P3CO) (15) (Fig. 2), and also by the vast majority of international publishers, including the American Society for Microbiology (ASM) and the Public Library of Science (PLOS) (Fig. 3). There are clearly experiments where the risks outweigh the benefits, and it is important that mechanisms exist to prevent such experiments. However, in many cases, gain-of-function research-of-concern can very clearly advance pandemic preparedness and the development of vaccines and antivirals. These tangible benefits often far outweigh the theoretical risks posed by modified viruses. Thus, it is important that oversight mechanisms faithfully consider both risks and benefits of these types of studies. It is equally important that the mechanisms used to provide oversight of gain-of-function research-of-concern be focused on research that is indeed of concern. Identifying research of concern is complex but it is critical that safeguards be both thoughtfully designed and implemented to avoid suppressing innovation in a field essential to mitigating infectious disease threats and with the potential to transform health.

### Existing regulation of virology research in the United States.

Without qualification, appropriate precautions should be taken to minimize laboratory accidents or the unjustified engineering of pathogens with enhanced pandemic potential. This is a shared goal for scientists and regulators. Virological research in the United States is subject to federal regulation through the Department of Health and Human Services and the U.S. Department of Agriculture for work involving human, animal, and plant pathogens. Federal policies are guided by the Biosafety in Microbiological and Biomedical Laboratories (BMBL) guidelines (16) published by the Centers for Disease Control and Prevention (CDC) and implemented by Institutional Biosafety Committees (IBCs) that manage the safety and practice of laboratory research at the local level (Fig. 1). The National Institutes of Health (NIH) established guidelines for the creation of IBCs in 1976 and updates these guidelines periodically. While each institution may have additional specific policies, the NIH requires that IBCs approve of research involving genetic manipulation of pathogens and that members of the local community are engaged through their inclusion on IBCs. As noted above, further oversight for research involving viruses with pandemic potential exists at multiple levels, including federal funding agencies (e.g., Department of Health and Human Services, Department of Defense), institutions (e.g., colleges, universities, and research institutes), and most accredited publishers of scientific research (Fig. 2 and 3) (17). It is important that we work to ensure appropriate and consistent implementation of these existing safeguards. We should also seek to develop international partnerships to ensure appropriate oversight is in place worldwide. While policy and infrastructure should frequently be reassessed with the goal of updating and improving biosafety and biosecurity, policy should not be changed rashly and without consideration of potential unintended consequences. Given the scientific complexities involved, such decisions should not be legislated. They should instead be made after full consideration by scientists with the relevant expertise – with appropriate oversight from Congress.

### Conclusion.

As we enter the fourth year of the COVID-19 pandemic, it is clear that the scientific establishment has been the most effective shield protecting humanity from this calamity through the delivery of rapid tests, vaccines, antibody therapies, and small molecule antivirals. Millions are alive today that would otherwise be dead thanks to society’s investment in creating and maintaining a vibrant scientific enterprise. In the debate on further restrictions around gain-of-function research and new regulations on virology in general, it is important to appreciate the existing framework of regulations at the federal and institutional levels. It is also critical to recognize that our ability to respond rapidly to emerging viral threats is dependent on our ability to apply the tools of modern biology to viruses. Regulations that are redundant with current practice or overly cumbersome will lead to unwarranted constraints on pandemic preparation and response and could leave humanity more vulnerable to future disease outbreaks. Gain-of-function research has been an extremely valuable tool in the development of vaccines and antivirals (Table 2). It already has layers of regulations and checks in place to ensure that potentially unsafe research is immediately reported (Fig. 2). It is critical that policy makers, virologists, and biosafety experts work together to ensure that research is conducted safely, with the common goal of reducing the burden of disease caused by viruses.
